# Laparoscopic repair of esophageal perforation following pneumatic dilatation for achalasia: a case series

**DOI:** 10.1093/jscr/rjag555

**Published:** 2026-07-06

**Authors:** Hoa Nguyen Xuan, Hieu Tong Quang, Phuc Chu Minh

**Affiliations:** Center for Gastrointestinal Surgery and Pelvic Floor Disorders, Viet Duc University Hospital, 40 Trang Thi Street, Hoan Kiem Ward, Hanoi 11024, Vietnam; Center for Gastrointestinal Surgery and Pelvic Floor Disorders, Viet Duc University Hospital, 40 Trang Thi Street, Hoan Kiem Ward, Hanoi 11024, Vietnam; Department of Hepato-biliary Surgery, Viet Duc University Hospital, 40 Trang Thi Street, Hoan Kiem Ward, Hanoi 11024, Vietnam

**Keywords:** achalasia, pneumatic dilation, esophageal perforation, Dor fundoplication, case report

## Abstract

Pneumatic dilation for achalasia is an effective treatment but carries a risk of esophageal perforation, a rare yet serious complication. We report two cases of esophageal perforation following pneumatic dilation for achalasia, both successfully managed using a laparoscopic transabdominal approach. The first was a 33-year-old woman who developed chest pain and dyspnea immediately after dilation, with imaging confirming a distal esophageal perforation and left pleural effusion. The second was a 30-year-old man who presented with chest pain two hours post-procedure, with computed tomography revealing a localized periesophageal fluid collection. Laparoscopic exploration identified full-thickness tears near the gastroesophageal junction, which were repaired primarily. Dor fundoplication, pleural drainage, and feeding jejunostomy were performed in both cases. Both patients tolerated the procedures well. Laparoscopic management is a safe and effective option for esophageal perforation after pneumatic dilation in carefully selected patients, particularly when the perforation is distal and diagnosed early.

## Introduction

Achalasia is a primary esophageal motility disorder of unknown etiology, characterized by impaired relaxation of the lower esophageal sphincter and abnormal esophageal peristalsis. The diagnosis of achalasia is typically based on two fundamental investigations: contrast esophagography and upper gastrointestinal endoscopy. On contrast studies, the classic finding is a ‘bird beak’ appearance at the gastroesophageal junction, often accompanied by esophageal dilation and abnormal motility. Endoscopy plays a crucial role in excluding secondary causes of esophageal obstruction, such as distal esophageal cancer or malignancy at the cardia [1, 2]. Achalasia is typically classified into three types. Type I (classic achalasia) is defined by minimal pressurization within the esophageal body. Type II (achalasia with esophageal compression), the most common subtype, is characterized by panesophageal pressurization in ˃20% of swallows. Type III (spastic achalasia) involves premature or spastic distal esophageal contractions, typically with more than two spastic segments [3].

Current treatment strategies for achalasia include endoscopic interventions, such as botulinum toxin injection, pneumatic dilation, and peroral endoscopic myotomy, and surgical management, most commonly laparoscopic Heller myotomy combined with an anti-reflux procedure. Each modality has its own advantages and limitations, although surgical myotomy is generally considered the first-line treatment. Pneumatic dilation is typically reserved for elderly patients, those with significant comorbidities that contraindicate surgery, or individuals who decline surgical intervention [4].

Pneumatic dilation is an endoscopic therapeutic approach in which the number of dilation sessions and duration of balloon inflation are tailored to the patient’s condition and the clinician’s experience. Complications are rare, with esophageal perforation being the most serious, occurring in ~2% of cases [5, 6]. Although perforation is a severe complication requiring prompt recognition and early management, standardized protocols for its treatment remain lacking in many centers.

## Case series

### Case 1

**Figure 1:**
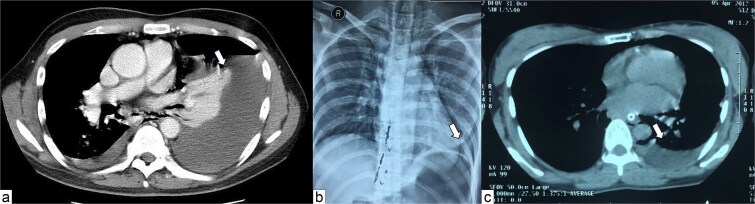
Chest X-ray and CT scan of the patients. (a) Marked pleural effusion (arrow) on contrast-enhanced CT scan of the first patient. (b) Chest X-ray findings of the second patient, note that there was unclear pleural effusion on initial X-ray (arrow). (c) Non-contrast CT findings of the second patient, with marked pleural effusion (arrow).

A 33-year-old female presented with a 4-year history of intermittent dysphagia. She was diagnosed with achalasia based on contrast esophagography and upper gastrointestinal endoscopy. The patient was scheduled for pneumatic dilation. During the procedure, a mucosal tear ~3 cm in length was identified just above the gastroesophageal junction. Following dilation, the patient developed chest pain and dyspnea. Chest radiography revealed a left-sided pleural effusion. Computed tomography (CT) of the chest and abdomen demonstrated a fluid collection adjacent to the left side of the distal esophagus above the cardia, along with left pleural effusion and no intra-abdominal fluid (Fig. 1a). Pleural ultrasonography confirmed left pleural effusion measuring 18 mm. Laboratory tests showed leukocytosis (12 × 10^9^/L).

The patient received early antibiotic therapy and underwent surgery 6 hours after dilation. A laparoscopic approach was performed, during which a 3 cm full-thickness esophageal tear was identified. Operative management included primary repair of the esophageal perforation, Dor fundoplication, left pleural drainage, and feeding jejunostomy. The operative time was 180 min, with negligible blood loss. Enteral feeding via jejunostomy was initiated 48 h postoperatively. A nasogastric tube was maintained on continuous low-pressure suction. On postoperative day (POD) 7, follow-up imaging was performed and the left pleural drain was removed. Esophageal contrast study on postoperative day 10 showed no evidence of leakage, and oral feeding was gradually resumed. The patient was discharged on POD 18. The patient has been followed annually for the next 2 years, with no complaint of dysphagia noted on follow-up visits. An esophago-gastro-duodenoscopy (EGD) after 2 years revealed complete healing of the tear (Fig. 4a).

### Case 2

A 30-year-old male presented with a 1-year history of intermittent dysphagia, which had progressively worsened over the preceding month. He was diagnosed with achalasia based on contrast esophagography and upper gastrointestinal endoscopy and was indicated for pneumatic dilation. No abnormalities were noted during the procedure. Two hours after dilation, the patient developed chest pain. Chest radiography was unremarkable (Fig. 1b); however, CT imaging of the chest and abdomen revealed a fluid collection adjacent to the left side of the distal esophagus above the cardia, without pleural or intra-abdominal fluid (Fig. 1c). Laboratory evaluation showed leukocytosis (15 × 10^9^/L).

**Figure 2:**
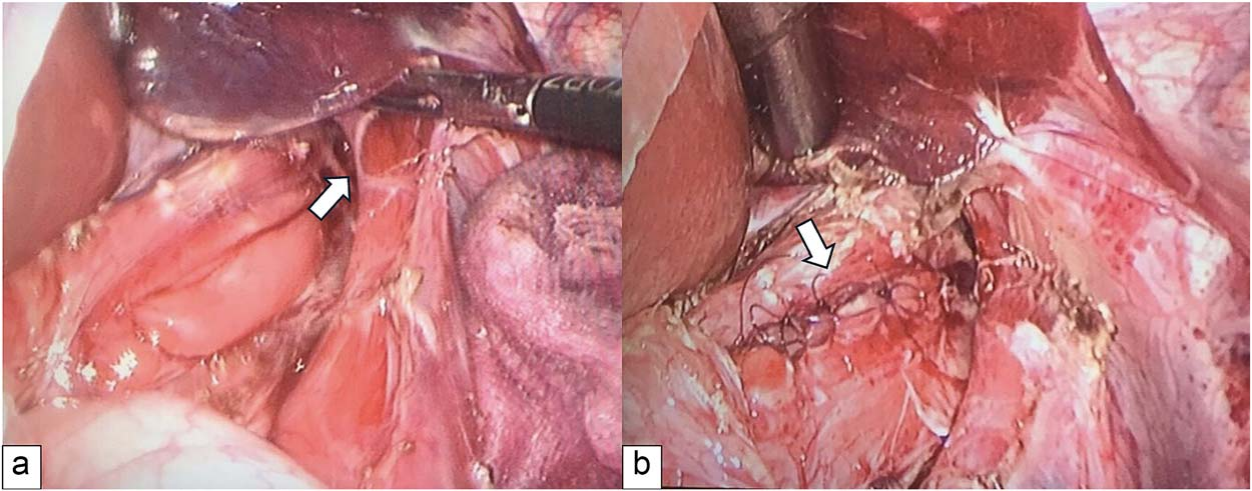
Intraoperative findings and management. (a) A 3.5 cm tear of the distal esophagus, as found upon laparoscopy (arrow). (b) Full-thickness repair (arrow).

The patient received prompt antibiotic therapy and underwent laparoscopic surgery 4 h after dilation. A 3.5 cm full-thickness esophageal tear was found upon laparoscopy (Fig. 2a). The procedure included primary repair of the esophageal perforation, Dor fundoplication, left pleural drainage, and feeding jejunostomy (Fig. 2b). The operative time was 160 min with minimal blood loss.

**Figure 3:**
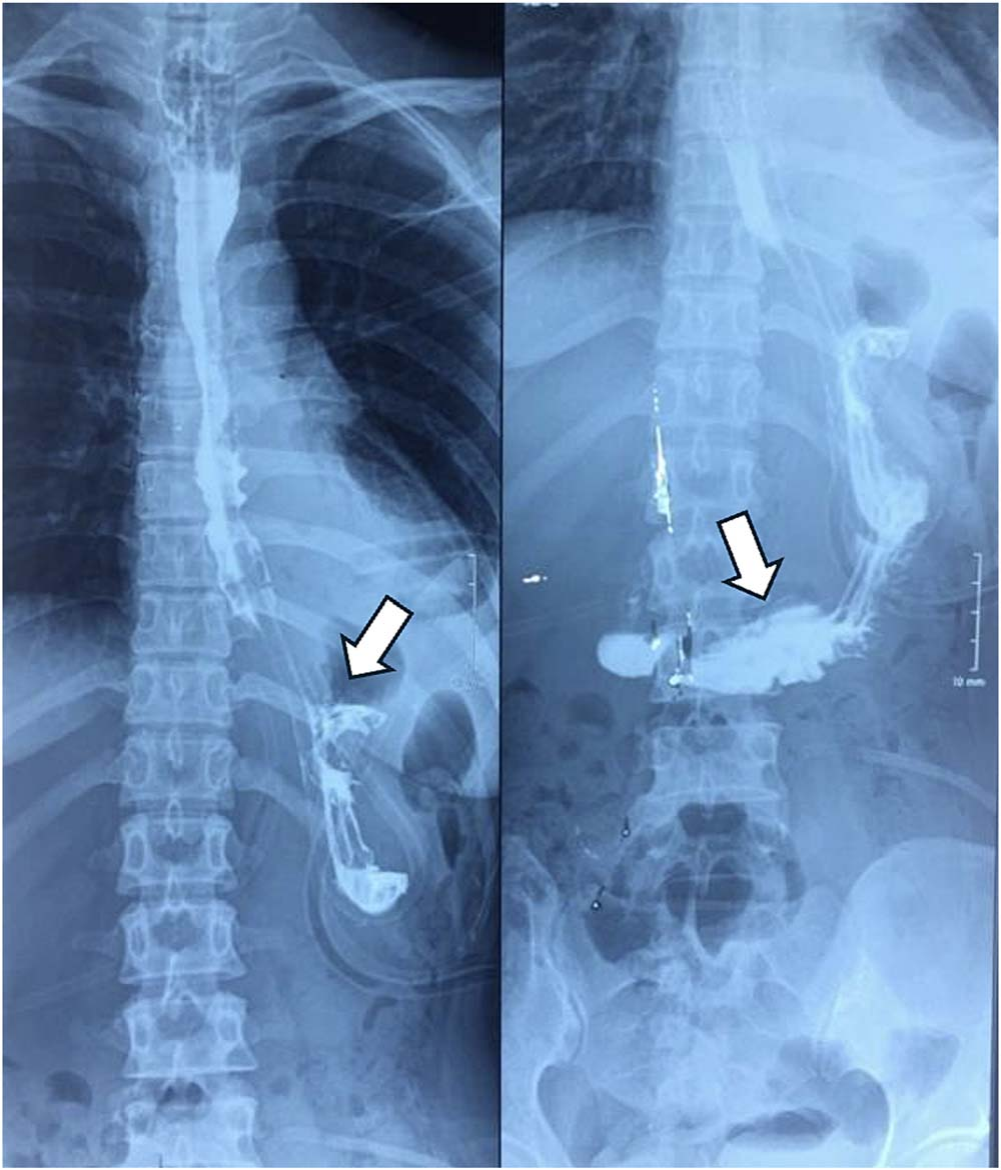
Postoperative contrast esophagogram. Normal esophago-gastric contrast flow was noted, with no leakage (arrow).

**Figure 4:**
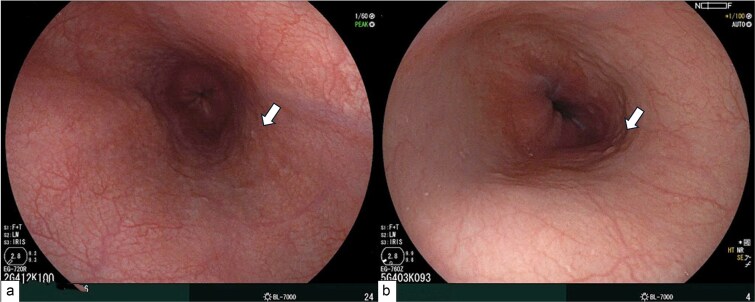
Follow-up EGD after 2 years. (a) EGD endoscopy findings of the first patient, with no visible scarring (arrow). (b) EGD endoscopy of the second patient, with minimal scarring (arrow).

Enteral feeding through the jejunostomy was initiated after 48 h. A nasogastric tube was placed for continuous low-pressure suction. On POD 5, follow-up ultrasonography revealed no pleural effusion and the left pleural drain was removed. Esophageal contrast study on POD 11 showed no leakage (Fig. 3a and b), and oral feeding was subsequently resumed. The patient was discharged on POD 21. There was no complaint of dysphagia during 2-year follow-up, and a subsequent EGD revealed complete healing of the tear with minimal scarring (Fig. 4b).

## Discussion

Esophageal perforation following pneumatic dilation most commonly occurs along the left wall of the distal esophagus, just above the lower esophageal sphincter [7, 8]. This region is anatomically more vulnerable due to relative muscular weakness, making it more susceptible to injury during dilation. Surgical repair is typically performed via either a left thoracic or transabdominal approach, with or without the addition of an anti-reflux procedure.

Clinical suspicion of esophageal perforation should be raised in patients presenting with severe or progressively worsening chest or abdominal pain following dilation. Fever may also be present. A definitive diagnosis is established when there is evidence of contrast leakage on contrast esophagogram into the mediastinum or peritoneal cavity, potentially leading to mediastinal abscess or peritonitis. In cases where contrast studies are inconclusive but clinical suspicion remains high, contrast-enhanced CT of the chest and abdomen, using both oral and intravenous contrast, should be performed to confirm the diagnosis.

Non-operative management depends on the extent and clinical presentation of the perforation. Selected patients with small leaks detected on contrast imaging and minimal or no clinical symptoms may be managed conservatively. Endoscopic approaches, such as clip placement for small mucosal tears, combined with nil per os (NPO), broad-spectrum antibiotics, and pleural drainage when indicated, have been reported [9–12].

However, these reports are limited by small sample sizes and relatively low success rates. The use of esophageal stents has also been described in isolated case reports, though evidence remains limited [13]. In general, treatment centers should be prepared to promptly manage esophageal perforation after dilation, with surgical planning to prevent progression to mediastinitis, pleural infection, or intra-abdominal sepsis. Delayed diagnosis may lead to severe complications, including septic shock, respiratory failure, and mediastinal abscess, making management significantly more challenging than primary repair [14]. There are two principal surgical approaches for repairing esophageal perforation.

Transabdominal approach is preferred for perforations located within 5 cm of the Z-line. It can be performed via open surgery or laparoscopy. The procedure involves primary repair of the mucosal and submucosal layers, typically combined with an anti-reflux procedure. Esophageal myotomy is generally not required, as the dilation itself often disrupts the muscular layer at the site of perforation [15]. However, some surgeons advocate full-thickness repair (including the muscle layer), in which case myotomy may be considered [7]. An anti-reflux procedure is recommended, making the abdominal approach advantageous. The choice of fundoplication depends on the location of the perforation and intraoperative findings. The gastric fundus can also be used as a patch to reinforce the repair. While Nissen fundoplication is widely used, partial fundoplication such as Dor (anterior) and Toupet (posterior) are also common. Although partial wraps may have higher rates of postoperative reflux compared to Nissen, some studies suggest that Nissen fundoplication may be associated with a higher long-term risk of recurrent dysphagia following esophageal myotomy [16].

Left thoracic approach is indicated for higher or posterior perforations that are difficult or impossible to access via the abdomen. A left thoracotomy through the 7th or 8th intercostal space allows direct repair of the mucosal and submucosal layers. In some cases, reinforcement using an intercostal muscle flap, with preservation of its vascular and nerve supply, may be performed. However, this technique is technically demanding due to the need for careful selection and preservation of the muscle flap [17, 18].

Although the thoracic approach has traditionally been the most common, our experience suggests that laparoscopic trans-hiatal access is highly effective for perforations located within 5 cm of the Z-line, with favorable outcomes. The laparoscopic abdominal approach offers several advantages over thoracic access. It facilitates the creation of an anti-reflux valve using the gastric fundus and provides a more familiar anatomical field for most surgeons. Additionally, laparoscopic mobilization allows gentle manipulation of the esophagus, improving exposure and making the perforation site more accessible.

One key limitation of this series is its small number of cases. Nevertheless, transabdominal laparoscopic repair of esophagus perforation following pneumatic dilation is feasible and reproducible for lesions ˂5 cm from the Z-line. The laparoscopic approach allows complete circumferential mobilization of the esophagus, precise identification and primary repair of the perforation, and, when necessary, esophageal myotomy and construction of an anti-reflux valve.
